# 2-Bromo­ethyl 2-chloro-6-methyl­quinoline-3-carboxyl­ate

**DOI:** 10.1107/S1600536810010160

**Published:** 2010-03-31

**Authors:** Saida Benzerka, Abdelmalek Bouraiou, Sofiane Bouacida, Thierry Roisnel, Ali Belfaitah

**Affiliations:** a Laboratoire des Produits Naturels d’Origine Végétale et de Synthèse Organique, PHYSYNOR, Université Mentouri-Constantine, 25000 Constantine, Algeria; b Unité de Recherche de Chimie de l’Environnement et Moléculaire Structurale, CHEMS, Université Mentouri-Constantine 25000, Algeria; c Centre de Difractométrie X, UMR 6226 CNRS Unité Sciences Chimiques de Rennes, Université de Rennes I, 263 Avenue du Général Leclerc, 35042 Rennes, France

## Abstract

In the title compound, C_13_H_11_BrClNO_2_, the two rings of the quinoline group are fused in an axial fashion at a dihedral angle of 1.28 (9)°. In the crystal, molecules are arranged in zigzag layers along the *c* axis. The crystal packing is stabilized by weak C—H⋯O hydrogen bonds and inter­molecular inter­actions between Br and O atoms [Br⋯O= 3.076 (2) Å], resulting in the formation of a three-dimensional network.

## Related literature

For our previous work on the preparation of quinoline derivatives, see: Benzerka *et al.* (2008 ▶); Ladraa *et al.* (2009 ▶, 2010 ▶). For radical bromination, see: Kikichi *et al.* (1998 ▶); Xu *et al.* (2003 ▶); Djerassi (1948 ▶); Newman & Lee (1972 ▶). For radical bromination of ketone and acetal functions, see: Marvell & Joncich (1951 ▶); Markees (1958 ▶).
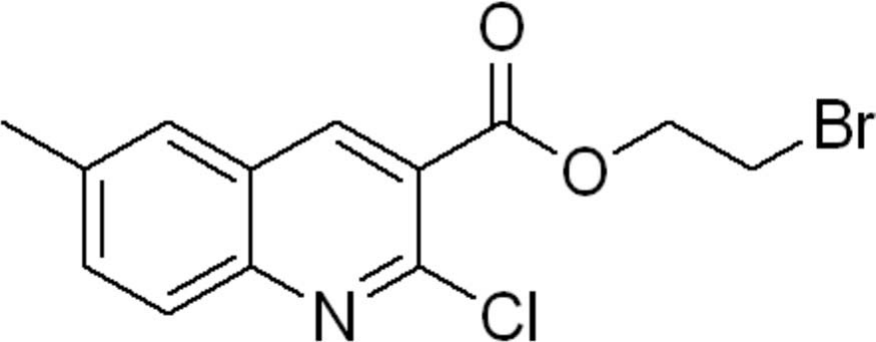


## Experimental

### 

#### Crystal data

C_13_H_11_BrClNO_2_
*M* *_r_* = 328.59
Monoclinic, 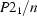

*a* = 6.1740 (4) Å
*b* = 29.0515 (14) Å
*c* = 7.2875 (4) Å
β = 99.167 (3)°
*V* = 1290.42 (13) Å^3^
*Z* = 4
Mo *K*α radiation
μ = 3.39 mm^−1^
*T* = 100 K
0.45 × 0.38 × 0.11 mm

#### Data collection

Bruker APEXII diffractometer
Absorption correction: multi-scan (*SADABS*; Sheldrick, 2002 ▶) *T*_min_ = 0.238, *T*_max_ = 0.689
11364 measured reflections
2938 independent reflections
2430 reflections with *I* > 2σ(*I*)
*R*_int_ = 0.054

#### Refinement

*R*[*F* ^2^ > 2σ(*F* ^2^)] = 0.037
*wR*(*F* ^2^) = 0.092
*S* = 1.02
2938 reflections
164 parameters
H-atom parameters constrained
Δρ_max_ = 0.74 e Å^−3^
Δρ_min_ = −0.85 e Å^−3^

### 

Data collection: *APEX2* (Bruker, 2001 ▶); cell refinement: *SAINT* (Bruker, 2001 ▶); data reduction: *SAINT*; program(s) used to solve structure: *SIR2002* (Burla *et al.*, 2005 ▶); program(s) used to refine structure: *SHELXL97* (Sheldrick, 2008 ▶); molecular graphics: *ORTEP-3 for Windows* (Farrugia, 1997 ▶) and *DIAMOND* (Brandenburg & Berndt, 2001 ▶); software used to prepare material for publication: *WinGX* (Farrugia, 1999 ▶).

## Supplementary Material

Crystal structure: contains datablocks global, I. DOI: 10.1107/S1600536810010160/bq2201sup1.cif

Structure factors: contains datablocks I. DOI: 10.1107/S1600536810010160/bq2201Isup2.hkl

Additional supplementary materials:  crystallographic information; 3D view; checkCIF report

## Figures and Tables

**Table 1:** Hydrogen-bond geometry (Å, °)

*D*—H⋯*A*	*D*—H	H⋯*A*	*D*⋯*A*	*D*—H⋯*A*
C13—H13*B*⋯O1^i^	0.97	2.41	3.347 (4)	162

Symmetry code: (i) 
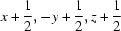
.

**Fig. 1.**
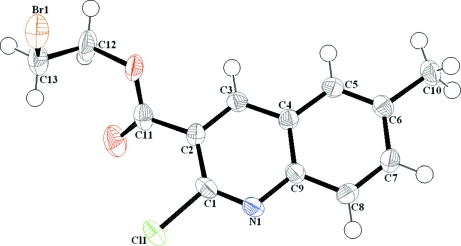
(Farrugia, 1997) the structure of the title compound with the atomic labelling scheme. Displacement are drawn at the 50% probability level.

**Fig. 2.**
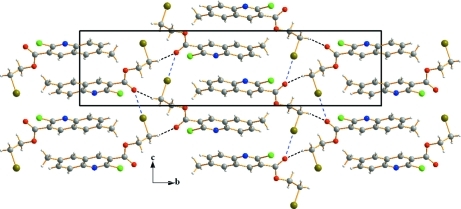
(Brandenburg & Berndt, 2001) Part of crystal packing of (I) showing hydrogen bond [C—H···O] and short interaction [Br···O]as dashed line.

**Fig. 3.**
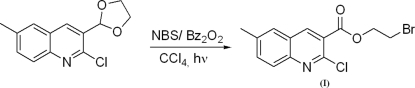
The formation of the title compound.

## supplementary crystallographic information

### Comment

Benzylic bromination can be carried out using *N*-bromosuccinimide (NBS) under photocatalytic conditions (Djerassi, 1948; Newman *et al.*, 1972). It is also known that NBS react with benzaldehyde diethylacetal to give corresponding ester (Marvell *et al.*, 1951; Markees *et al.*, 1958). Although extensive studies have been carried out in the past, selectivity clearly remains a common problem in radical bromination (Kikichi *et al.*, 1998; Xu *et al.*, 2003). In previous works, we have reported structure determination of some new quinoline derivatives (Benzerka *et al.*, 2008; Ladraa *et al.*, 2009; Ladraa *et al.*, 2010). In this paper, we report the synthesis and structure determination of new compound, resulting from the radical bromination of 2-chloro-3-(1,3-dioxolan-2-yl)-6-methylquinoline, (I), under photocatalytic conditions. Our attempt to brominate the methyl group linked at C-6 position of quinoline ring, which has an acetal function at C-3, was failed and led to the 2-bromoethyl 2-chloro-6-methylquinoline-3-carboxylate (I). This compound is the result of the unwanted conversion of the acetal to the corresponding ester.

The molecular geometry and the atom-numbering scheme of (I) are shown in Figure 1. The asymmetric unit of title molecule contains a 2-bromoethylcarboxylate group linked to quinolyl moiety. The two rings of quinolyl moiety are fused in an axial fashion and form a dihedral angle of 1.28 (9)° The crystal structure can be described as layers in zig zag along of c-axis which quinoline rings are parallel to the (110) plane. The crystal packing is stabilized by weak hydrogen bonds [C—H···O] and intermolecular interactions between Br and O atoms [Br···O= 3.076 (2)] (Figure 2), resulting in the formation of a three dimensional network and reinforcing a cohesion of structure. Hydrogen-bonding parameters are listed in Table 1.

### Experimental

The title compound (I) was synthesized by treating 1 mmol. of 2-chloro-3-(1,3-dioxolan-2-yl)-6-methylquinoline with 1 mmol. of *N*-bromosuccinimide in the presence of 0.5 mmol. of dibenzoylperoxide in CCl4 under photocatalytic conditions. The contents were then cooled and filtered off and the filtrate was concentrated under reduced pressure. The residue was subjected to column chromatography (silica gel, eluent: CH2Cl2) to afford pure product. Crystals suitable for x-ray analysis were obtained by slow evaporation of a dichloromethane solution of (I).

### Refinement

All H atoms were localized on Fourier maps but introduced in calculated positions and treated as riding on their parent C atom. (with C—H = 0.93Å, 0.96Å, 0.97Å and U_iso_(H) =1.2 or 1.5(carrier atom)).

### Crystal data

**Table:**

C_13_H_11_BrClNO_2_	*F*(000) = 656
*M**_r_* = 328.59	*D*_x_ = 1.691 Mg m^−^^3^
Monoclinic, *P*2_1_/*n*	Mo *K*α radiation, λ = 0.71073 Å
*a* = 6.1740 (4) Å	Cell parameters from 3765 reflections
*b* = 29.0515 (14) Å	θ = 2.8–27.3°
*c* = 7.2875 (4) Å	µ = 3.39 mm^−^^1^
β = 99.167 (3)°	*T* = 100 K
*V* = 1290.42 (13) Å^3^	Prism, colourless
*Z* = 4	0.45 × 0.38 × 0.11 mm

### Data collection

**Table:**

Bruker APEXII diffractometer	2938 independent reflections
Radiation source: Enraf–Nonius FR590	2430 reflections with *I* > 2σ(*I*)
graphite	*R*_int_ = 0.054
CCD rotation images, thick slices scans	θ_max_ = 27.5°, θ_min_ = 2.8°
Absorption correction: multi-scan (*SADABS*; Sheldrick, 2002)	*h* = −7→7
*T*_min_ = 0.238, *T*_max_ = 0.689	*k* = −37→37
11364 measured reflections	*l* = −9→9

### Refinement

**Table:**

Refinement on *F*^2^	Primary atom site location: structure-invariant direct methods
Least-squares matrix: full	Secondary atom site location: difference Fourier map
*R*[*F*^2^ > 2σ(*F*^2^)] = 0.037	Hydrogen site location: inferred from neighbouring sites
*wR*(*F*^2^) = 0.092	H-atom parameters constrained
*S* = 1.02	*w* = 1/[σ^2^(*F*_o_^2^) + (0.0397*P*)^2^ + 1.2764*P*] where *P* = (*F*_o_^2^ + 2*F*_c_^2^)/3
2938 reflections	(Δ/σ)_max_ = 0.001
164 parameters	Δρ_max_ = 0.74 e Å^−^^3^
0 restraints	Δρ_min_ = −0.85 e Å^−^^3^

### Special details

**Table:**

Geometry. All esds (except the esd in the dihedral angle between two l.s. planes) are estimated using the full covariance matrix. The cell esds are taken into account individually in the estimation of esds in distances, angles and torsion angles; correlations between esds in cell parameters are only used when they are defined by crystal symmetry. An approximate (isotropic) treatment of cell esds is used for estimating esds involving l.s. planes.
Refinement. Refinement of *F*^2^ against ALL reflections. The weighted *R*-factor wR and goodness of fit *S* are based on *F*^2^, conventional *R*-factors *R* are based on *F*, with *F* set to zero for negative *F*^2^. The threshold expression of *F*^2^ > σ(*F*^2^) is used only for calculating *R*-factors(gt) etc. and is not relevant to the choice of reflections for refinement. *R*-factors based on *F*^2^ are statistically about twice as large as those based on *F*, and *R*- factors based on ALL data will be even larger.

### Fractional atomic coordinates and isotropic or equivalent isotropic displacement parameters (Å^2^)

**Table:**

	*x*	*y*	*z*	*U*_iso_*/*U*_eq_	
C1	0.7808 (4)	0.08966 (9)	0.2084 (4)	0.0164 (5)	
C2	1.0027 (4)	0.10023 (8)	0.2862 (4)	0.0152 (5)	
C3	1.1423 (4)	0.06355 (9)	0.3242 (4)	0.0151 (5)	
H3	1.2874	0.0687	0.3775	0.018*	
C4	1.0694 (4)	0.01828 (9)	0.2837 (4)	0.0147 (5)	
C5	1.2083 (4)	−0.02083 (9)	0.3149 (4)	0.0167 (6)	
H5	1.3551	−0.0169	0.3657	0.02*	
C6	1.1299 (4)	−0.06439 (9)	0.2713 (4)	0.0161 (5)	
C7	0.9057 (5)	−0.06973 (9)	0.1919 (4)	0.0180 (6)	
H7	0.8517	−0.0991	0.1616	0.022*	
C8	0.7665 (4)	−0.03275 (9)	0.1586 (4)	0.0181 (6)	
H8	0.6206	−0.0371	0.1057	0.022*	
C9	0.8464 (4)	0.01207 (9)	0.2053 (4)	0.0147 (5)	
C10	1.2752 (5)	−0.10631 (9)	0.3046 (4)	0.0204 (6)	
H10A	1.425	−0.0968	0.3394	0.031*	
H10B	1.2613	−0.1244	0.193	0.031*	
H10C	1.2317	−0.1244	0.4027	0.031*	
C11	1.0855 (4)	0.14836 (9)	0.3158 (4)	0.0188 (6)	
C12	1.3409 (5)	0.19567 (9)	0.5055 (5)	0.0263 (7)	
H12A	1.3736	0.2075	0.3885	0.032*	
H12B	1.4788	0.1915	0.5883	0.032*	
C13	1.2040 (5)	0.23018 (9)	0.5886 (5)	0.0247 (7)	
H13A	1.065	0.2342	0.507	0.03*	
H13B	1.2789	0.2596	0.6002	0.03*	
N1	0.7035 (4)	0.04864 (7)	0.1709 (3)	0.0164 (5)	
O1	1.0343 (4)	0.17987 (7)	0.2112 (3)	0.0292 (5)	
O2	1.2320 (3)	0.15146 (6)	0.4733 (3)	0.0199 (4)	
Cl1	0.58811 (11)	0.13419 (2)	0.16345 (10)	0.02179 (17)	
Br1	1.15019 (5)	0.209908 (9)	0.83386 (5)	0.02814 (11)	

### Atomic displacement parameters (Å^2^)

**Table:**

	*U*^11^	*U*^22^	*U*^33^	*U*^12^	*U*^13^	*U*^23^
C1	0.0161 (13)	0.0178 (12)	0.0153 (13)	0.0059 (10)	0.0026 (11)	0.0012 (11)
C2	0.0163 (13)	0.0125 (11)	0.0176 (13)	−0.0006 (9)	0.0050 (11)	−0.0013 (10)
C3	0.0117 (12)	0.0159 (12)	0.0180 (13)	0.0001 (9)	0.0030 (11)	0.0004 (11)
C4	0.0134 (12)	0.0140 (11)	0.0169 (13)	−0.0012 (9)	0.0035 (11)	0.0000 (10)
C5	0.0124 (12)	0.0174 (12)	0.0200 (14)	0.0012 (10)	0.0018 (11)	−0.0007 (11)
C6	0.0177 (13)	0.0160 (12)	0.0150 (13)	0.0031 (9)	0.0039 (11)	0.0005 (11)
C7	0.0207 (14)	0.0130 (12)	0.0198 (14)	−0.0013 (10)	0.0019 (12)	−0.0006 (11)
C8	0.0151 (13)	0.0179 (12)	0.0202 (14)	−0.0014 (10)	−0.0004 (11)	−0.0036 (11)
C9	0.0147 (13)	0.0142 (12)	0.0151 (13)	0.0016 (9)	0.0017 (11)	0.0002 (10)
C10	0.0207 (14)	0.0147 (12)	0.0261 (16)	0.0042 (10)	0.0045 (12)	−0.0006 (11)
C11	0.0169 (13)	0.0144 (12)	0.0281 (16)	0.0005 (10)	0.0123 (12)	−0.0014 (12)
C12	0.0254 (15)	0.0119 (12)	0.043 (2)	−0.0056 (11)	0.0098 (14)	−0.0054 (13)
C13	0.0291 (16)	0.0125 (12)	0.0335 (18)	−0.0003 (11)	0.0079 (14)	−0.0005 (12)
N1	0.0133 (11)	0.0173 (10)	0.0179 (12)	0.0036 (8)	0.0005 (9)	−0.0005 (10)
O1	0.0349 (12)	0.0158 (9)	0.0376 (13)	0.0016 (8)	0.0084 (11)	0.0066 (10)
O2	0.0208 (10)	0.0117 (8)	0.0280 (11)	−0.0022 (7)	0.0062 (9)	−0.0040 (8)
Cl1	0.0196 (3)	0.0190 (3)	0.0273 (4)	0.0092 (2)	0.0050 (3)	0.0016 (3)
Br1	0.03431 (19)	0.01765 (15)	0.0334 (2)	0.00054 (11)	0.00824 (14)	−0.00443 (13)

### Geometric parameters (Å, °)

**Table:**

C1—N1	1.296 (3)	C8—C9	1.415 (3)
C1—C2	1.430 (4)	C8—H8	0.93
C1—Cl1	1.753 (3)	C9—N1	1.378 (3)
C2—C3	1.371 (3)	C10—H10A	0.96
C2—C11	1.493 (3)	C10—H10B	0.96
C3—C4	1.406 (3)	C10—H10C	0.96
C3—H3	0.93	C11—O1	1.201 (3)
C4—C9	1.416 (4)	C11—O2	1.346 (3)
C4—C5	1.420 (3)	C12—O2	1.451 (3)
C5—C6	1.374 (4)	C12—C13	1.500 (4)
C5—H5	0.93	C12—H12A	0.97
C6—C7	1.422 (4)	C12—H12B	0.97
C6—C10	1.509 (3)	C13—Br1	1.960 (3)
C7—C8	1.373 (4)	C13—H13A	0.97
C7—H7	0.93	C13—H13B	0.97
			
N1—C1—C2	125.3 (2)	N1—C9—C4	121.9 (2)
N1—C1—Cl1	115.0 (2)	C8—C9—C4	119.6 (2)
C2—C1—Cl1	119.6 (2)	C6—C10—H10A	109.5
C3—C2—C1	116.4 (2)	C6—C10—H10B	109.5
C3—C2—C11	120.6 (2)	H10A—C10—H10B	109.5
C1—C2—C11	122.9 (2)	C6—C10—H10C	109.5
C2—C3—C4	121.0 (2)	H10A—C10—H10C	109.5
C2—C3—H3	119.5	H10B—C10—H10C	109.5
C4—C3—H3	119.5	O1—C11—O2	124.3 (2)
C3—C4—C9	117.5 (2)	O1—C11—C2	125.0 (3)
C3—C4—C5	123.4 (2)	O2—C11—C2	110.7 (2)
C9—C4—C5	119.1 (2)	O2—C12—C13	112.4 (2)
C6—C5—C4	121.2 (2)	O2—C12—H12A	109.1
C6—C5—H5	119.4	C13—C12—H12A	109.1
C4—C5—H5	119.4	O2—C12—H12B	109.1
C5—C6—C7	118.6 (2)	C13—C12—H12B	109.1
C5—C6—C10	121.9 (2)	H12A—C12—H12B	107.9
C7—C6—C10	119.5 (2)	C12—C13—Br1	110.8 (2)
C8—C7—C6	121.9 (2)	C12—C13—H13A	109.5
C8—C7—H7	119.1	Br1—C13—H13A	109.5
C6—C7—H7	119.1	C12—C13—H13B	109.5
C7—C8—C9	119.5 (2)	Br1—C13—H13B	109.5
C7—C8—H8	120.2	H13A—C13—H13B	108.1
C9—C8—H8	120.2	C1—N1—C9	117.9 (2)
N1—C9—C8	118.5 (2)	C11—O2—C12	115.3 (2)
			
N1—C1—C2—C3	−0.3 (4)	C3—C4—C9—N1	−0.6 (4)
Cl1—C1—C2—C3	177.6 (2)	C5—C4—C9—N1	−179.7 (3)
N1—C1—C2—C11	176.2 (3)	C3—C4—C9—C8	178.7 (3)
Cl1—C1—C2—C11	−5.9 (4)	C5—C4—C9—C8	−0.4 (4)
C1—C2—C3—C4	1.4 (4)	C3—C2—C11—O1	137.8 (3)
C11—C2—C3—C4	−175.2 (3)	C1—C2—C11—O1	−38.6 (4)
C2—C3—C4—C9	−1.0 (4)	C3—C2—C11—O2	−40.1 (4)
C2—C3—C4—C5	178.1 (3)	C1—C2—C11—O2	143.5 (3)
C3—C4—C5—C6	−179.3 (3)	O2—C12—C13—Br1	62.8 (3)
C9—C4—C5—C6	−0.3 (4)	C2—C1—N1—C9	−1.2 (4)
C4—C5—C6—C7	0.6 (4)	Cl1—C1—N1—C9	−179.2 (2)
C4—C5—C6—C10	−179.6 (3)	C8—C9—N1—C1	−177.6 (3)
C5—C6—C7—C8	−0.3 (4)	C4—C9—N1—C1	1.7 (4)
C10—C6—C7—C8	−180.0 (3)	O1—C11—O2—C12	−4.3 (4)
C6—C7—C8—C9	−0.4 (4)	C2—C11—O2—C12	173.6 (2)
C7—C8—C9—N1	−179.9 (3)	C13—C12—O2—C11	81.8 (3)
C7—C8—C9—C4	0.8 (4)		

### Hydrogen-bond geometry (Å, °)

**Table:**

*D*—H···*A*	*D*—H	H···*A*	*D*···*A*	*D*—H···*A*
C13—H13B···O1^i^	0.97	2.41	3.347 (4)	162

Symmetry codes: (i) *x*+1/2, −*y*+1/2, *z*+1/2.
